# Evolving Perspective on the Origin and Diversification of Cellular Life and the Virosphere

**DOI:** 10.1093/gbe/evac034

**Published:** 2022-02-26

**Authors:** Anja Spang, Tara A Mahendrarajah, Pierre Offre, Courtney W Stairs

**Affiliations:** Department of Marine Microbiology and Biogeochemistry, NIOZ, Royal Netherlands Institute for Sea Research, Utrecht University, Den Burg, The Netherlands; Department of Cell and Molecular Biology, Science for Life Laboratory, Uppsala University, Uppsala, Sweden; Department of Marine Microbiology and Biogeochemistry, NIOZ, Royal Netherlands Institute for Sea Research, Utrecht University, Den Burg, The Netherlands; Department of Marine Microbiology and Biogeochemistry, NIOZ, Royal Netherlands Institute for Sea Research, Utrecht University, Den Burg, The Netherlands; Department of Biology, Microbiology research group, Lund University, Lund, Sweden

**Keywords:** tree of life, viruses, archaea, bacteria and eukaryotes, eukaryogenesis, diversity and evolution, methodological progress

## Abstract

The tree of life (TOL) is a powerful framework to depict the evolutionary history of cellular organisms through time, from our microbial origins to the diversification of multicellular eukaryotes that shape the visible biosphere today. During the past decades, our perception of the TOL has fundamentally changed, in part, due to profound methodological advances, which allowed a more objective approach to studying organismal and viral diversity and led to the discovery of major new branches in the TOL as well as viral lineages. Phylogenetic and comparative genomics analyses of these data have, among others, revolutionized our understanding of the deep roots and diversity of microbial life, the origin of the eukaryotic cell, eukaryotic diversity, as well as the origin, and diversification of viruses. In this review, we provide an overview of some of the recent discoveries on the evolutionary history of cellular organisms and their viruses and discuss a variety of complementary techniques that we consider crucial for making further progress in our understanding of the TOL and its interconnection with the virosphere.

**Significance**

Our review provides a timely overview of how recent methodological progress has allowed an updated view on the tree of life and its connection to the virosphere. It covers topics ranging from last universal common ancestor to last eukaryotic common ancestor and the extant diversity of prokaryotic and eukaryotic life as well as viruses. Furthermore, we summarize current developments in the field that can help to make further progress in our understanding of deep evolution in the coming years.

## Introduction

**Fig. 1.**
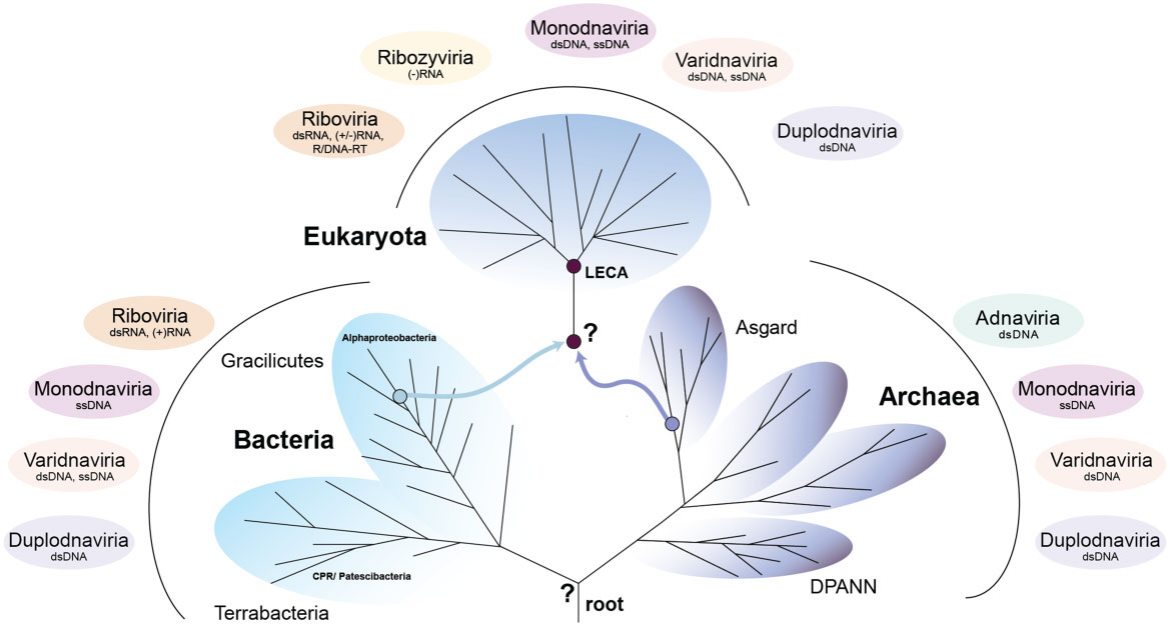
Tree of cellular life (TOL) and connection to the six major realms of viruses. The tree is a schematic representation of the relationship of the major domains of life, comprised of the primary domains of Archaea and Bacteria and the secondary domain of Eukaryota. The assumption that Archaea and Bacteria form separate domains of life is dependent on the placement of the root between those domains, though this hypothesis remains to be validated. Although the node separating the DPANN (acronym referenced in text) from all other archaeal clades has been suggested to be the most ancestral split on the archaeal branch, the CPR (acronym referenced in text) most likely represents a more recently evolved sister-clade of the Chloroflexota (Coleman et al. 2021). Current data support an origin of the eukaryotic cell through a symbiosis between an ancestral member of the Asgard archaea (also Asgardarchaeota) (purple arrow) and Alphaproteobacteria (blue arrow), though the timing of the mitochondrial acquisition is debated and the events leading to LECA are poorly resolved. On the outside of the TOL, we illustrate the connection of the three cellular domains with virus representatives belonging to either of the six major viral realms, the Riboviria, Monodnaviria, Varidnaviria, Duplodnaviria, Adnaviria, and Ribozyviria (Krupovic et al. 2020; Koonin et al. 2021). The latter two realms are restricted to the Archaea or eukaryotes, respectively. The Riboviria have so far only been found associated with Bacteria and eukaryotes, whereas all other realms include members infecting cellular organisms across the TOL. LECA, last eukaryotic common ancestor.

All cellular life forms (organisms) on Earth can be assigned to one of the major domains—the Archaea, Bacteria, or Eukaryota (hereafter referred to as eukaryotes) (Woese and Fox 1977; Woese et al. 1990). Because all organisms have evolved from a shared last universal common ancestor (LUCA) (Weiss et al. 2016), the relationship of extant organisms is often depicted within the framework of a tree of life (TOL) (Dagan et al. 2008; Puigbò et al. 2009; Blais and Archibald 2021). Upon the discovery of the Archaea, it was assumed that the TOL comprises three distinct branches that evolved vertically since LUCA, with the Bacteria on one side of the root and Archaea and eukaryotes forming sister clades on the other side of the root (Woese et al. 1990). However, recent years have witnessed an increasing body of evidence suggesting that eukaryotes, which comprise both uni- and multicellular representatives, have emerged through a symbiosis of an archaeon and a bacterium, that is, through the merging of two branches from within the Archaea and Bacteria, respectively (fig. 1) (Guy et al. 2014; Koonin and Yutin 2014; Martin et al. 2015; Eme et al. 2017; Lopez-Garcia and Moreira 2020). In turn, Archaea and Bacteria are often referred to as primary domains of life while eukaryotes form a secondary domain of life (Williams et al. 2013, 2020). In contrast, viruses are noncellular obligate intracellular parasites that infect all cellular life forms (Koonin and Starokadomskyy 2016). Similar to other selfish genetic elements, viruses are generally not considered within the framework of the TOL (Moreira and Lopez-Garcia 2009), but are an integral part of the biosphere or biological realm (Koonin and Starokadomskyy 2016). They also impact genome evolution of cellular life not only through the exchange of genes with their hosts but also through host-parasite coevolution (Popa and Dagan 2011; Koonin 2016). In fact, the prevalence of horizontal gene transfer (HGT) via both mobile genetic elements (MGEs) and viruses but also directly between distinct organisms has to some extent questioned the concept of a TOL, which may be more correctly represented as a network including both vertical and horizontal branches (Doolittle and Bapteste 2007; Dagan et al. 2008; Puigbò et al. 2009). Yet, despite this component of horizontal genome evolution, the “statistical” TOL has remained a useful concept for understanding life’s diversification (Koonin 2015b; Blais and Archibald 2021).

Recently, the application of cultivation-independent metagenomic and single-cell genomic techniques has improved our knowledge of microbial and viral diversity and, in turn, our view of the TOL (Hug et al. 2016) and its connection to the virosphere (Krupovic et al. 2020). For example, during the past decade a plethora of previously unknown archaeal and bacterial taxa (e.g., reviewed in Adam et al. [2017]; Spang et al. [2017]; Castelle and Banfield [2018]) have been described, including various lineages of high-taxonomic rank at the phylum and class-level (Hug et al. 2016; Parks et al. 2018; Rinke et al. 2021). Furthermore, progress has been made with regard to our understanding of the origin of eukaryotes (Eme et al. 2017) as well as their subsequent diversification (Burki et al. 2020). Genomics approaches have also transformed our knowledge on the vast diversity of viruses (Paez-Espino et al. 2016; Martinez-Hernandez et al. 2017; Gregory et al. 2019; Beaulaurier et al. 2020; Moniruzzaman, Martinez-Gutierrez et al. 2020; Bellas and Sommaruga 2021; Edgar et al. 2022), their putative host taxa (Roux et al. 2015; Dzunkova et al. 2019; Jarett et al. 2020; Sakowski et al. 2021), and origins (Krupovic et al. 2019).

In the following, we will provide an updated perspective of the TOL and virosphere by focusing on selected key findings. Furthermore, we describe a variety of research approaches, which we consider important for making further progress on our understanding of the history of life on Earth.

## The Primary Domains of Life and Deep Roots of the TOL

The nature of LUCA and the emergence of the two primary domains of life are some of the most fundamental unknowns in our understanding of life’s evolution. Archaeal and bacterial cells are distinguished by major differences in their cell lipid membrane and use of contrasting molecular machinery, including for the replication, and processing of genetic information. Although a wide variety of hypotheses have been proposed to explain the distinct cell membranes of bacteria and archaea and the early evolution of their metabolism, these remain controversial and progress has been constrained by the limited availability of relevant data (Schoepp-Cothenet et al. 2013; Sousa et al. 2013; Sojo et al. 2014; Russell and Nitschke 2017). It is generally assumed that the root in the TOL separates Archaea and Bacteria as inferred based on the use of ancient paralogous gene families for rooting (Iwabe et al. 1989; Brown and Doolittle 1995; Zhaxybayeva et al. 2005; Weinheimer and Aylward 2020) and genome networks (Dagan et al. 2010) (fig. 1). Yet, the accurate placement of the root is challenging and prone to phylogenetic artifacts and alternative roots, such as within Bacteria (Cavalier-Smith 2006; Lake et al. 2009), have not been formally ruled out (Gouy et al. 2015). Further, it has recently been suggested that the branch separating the primary domains of life may be shorter than in previous estimates (Zhu et al. 2019). However, it was subsequently shown that the reduced estimate of the Archaea/Bacteria branch length most likely results from inter-domain gene transfers and, in agreement with earlier work (Koonin 2015b; Hug et al. 2016), that the longest branch in the TOL lies between Archaea and Bacteria (Martinez-Gutierrez and Aylward 2021; Moody et al. 2022) (note that these analyses did not consider extremely fast-evolving symbionts and parasites). Improved phylogenetic models, the integration of genomic data from the diversity of recently discovered taxa as well as the use of novel approaches for rooting, such as gene tree-species tree reconciliations, for example, Szöllõsi et al. (2012), David and Alm (2011), and Szöllõsi et al. (2013)  (see below), will help to determine whether this branch indeed represents the deepest split in the TOL.

Particularly, the discovery of two previously unknown and potentially deep-branching microbial radiations in the Bacteria and Archaea, the so-called DPANN archaea (Rinke et al. 2013; Castelle et al. 2015) and the bacterial Candidate Phyla Radiation (CPR or Patescibacteria) (Brown et al. 2015), respectively, has provided important data for readdressing the deep roots of microbial life and the placement of the archaeal and bacterial roots (Williams et al. 2017; Castelle et al. 2018; Taib et al. 2020; Coleman et al. 2021; Xavier et al. 2021). The DPANN group (acronym referring to its first described member lineages, the Diapherotrites, Parv-, Aenigm-, Nano-, and Nanohaloarchaeota) now includes more than eight distinct archaeal phyla (Rinke et al. 2021) that group together with Nanoarchaeota, an archaeal clade represented by the ultrasmall and ectosymbiotic archaeon *Nanoarchaeum equitans* (Huber et al. 2002). Representatives of DPANN have small genomes and cell sizes, are characterized by restricted anabolic and catabolic capabilities, and include obligate ectosymbionts some of which have been cultivated in coculture with their hosts belonging to the Halobacteriota, Thermoproteota, and Thermoplasmatota (Huber et al. 2002; Podar et al. 2013; Munson-McGee et al. 2015; Wurch et al. 2016; Golyshina et al. 2017; Krause et al. 2017; Hamm et al. 2019; St John et al. 2019; La Cono et al. 2020; Sakai et al. 2022). Indeed, symbiotic lifestyles have been suggested to represent a common feature of genome-reduced members of the DPANN (Castelle et al. 2018). Likewise, members of the CPR, which also include various lineages of high taxonomic rank, share several genomic features with the DPANN archaea, such as small cell and genome sizes, a limited metabolic potential and potential dependency on partner organisms (Castelle et al. 2018). In line with this, two representatives of this group, that is, members of the Saccharibacteria and Absconditabacteria, have been successfully enriched as symbionts in coculture with their respective actinobacterial and gammaproteobacterial hosts (He et al. 2015; Bor et al. 2018; Moreira et al. 2021). It seems that the level of host specificity differs significantly between different representatives of the DPANN and CPR. For instance, although the most genome-reduced members of the DPANN, such as *N. equitans*, seem unable to switch between different host strains (Jahn et al. 2008), members of the Micrarchaeota infect hosts belonging to different archaeal phyla and comprise strains that can grow in coculture with hosts belonging to different genera (Golyshina et al. 2017; Krause et al. 2017; Sakai et al. 2022). Furthermore, it seems that at least DPANN may also include free-living members such as the Altiarchaeota (Probst et al. 2014) or members, which, in spite of certain auxotrophies, do not require permanent physical contact with potentially interacting partners (Youssef et al. 2015; Beam et al. 2020).

Initial phylogenetic analyses have recovered both the CPR (Brown et al. 2015) and DPANN (Rinke et al. 2013; Castelle et al. 2015) as monophyletic and early diverging branches in the TOL (fig. 1), but these findings are being debated (Dombrowski et al. 2019; Meheust et al. 2019). In particular, several authors have raised the concern, that the deep and monophyletic placement of DPANN and CPR lineages may be the result of phylogenetic artifacts (Brochier-Armanet et al. 2011; Petitjean et al. 2014; Raymann et al. 2014; Aouad et al. 2018; Feng et al. 2021) such as long-branch attraction, that leads to the erroneous grouping of fast-evolving taxa in a monophyletic clade as well as their attraction to a distant outgroup (Bergsten 2005; Philippe et al. 2005). For example, previous studies have revealed that genomes of other symbionts (e.g., obligate intracellular bacterial endosymbionts) indeed experience faster evolutionary rates, have compositional biases and form long branches in phylogenetic trees (Moran 1996; Rodriguez-Brito et al. 2006). In turn, elucidating the phylogenetic placement of the symbiotic CPR and DPANN has proven challenging and requires careful phylogenetic approaches implementing, among others, careful marker gene and taxon selection approaches and/or the use of complex models of evolution that account for differences in evolutionary rates across sites and lineages (Dombrowski et al. 2020; Coleman et al. 2021; Martinez-Gutierrez and Aylward 2021). Furthermore, such analyses benefit from taking into account potentially increased rates of HGT between symbionts and their hosts (Dombrowski et al. 2020).

Recently, outgroup-free rooting methods have been applied to assess the placement of CPR and DPANN in the TOL. For instance, Coleman et al. (2021) have used a gene tree—species tree reconciliation approach (Szöllõsi et al. 2012; David and Alm 2011; Szöllõsi et al. 2013) to root the bacterial tree and reconstruct the proteome of the last bacterial common ancestor. Interestingly, and in contrast to several earlier studies, this has revealed that the CPR most likely represents a more recently evolved monophyletic sister-lineage of the Chloroflexota (Coleman et al. 2021) rather than an early diverged bacterial clade (Brown et al. 2015) (fig. 1). Thus, CPR members seem to be derived from more complex ancestors with their small genomes being a result of genome-streamlining processes (Coleman et al. 2021). In agreement with this, a recent analysis aiming to resolve the evolution of cell envelopes in Bacteria not only indicated the ancestry of didermy with several independent transitions to monoderm phenotypes but also supported a sisterhood relationship of Chloroflexota and CPR nested within Terrabacteria (Taib et al. 2020). Finally, the careful assessment of marker genes for multidomain phylogenies has further confirmed this derived placement of the CPR (Martinez-Gutierrez and Aylward 2021).

In contrast, several recent studies have provided support for the “clanhood” of DPANN in unrooted phylogenies, their characteristic set of genes and their placement as an early radiation on the archaeal branch of the TOL raising the possibility that DPANN clades may have evolved in parallel with their host lineages over much of evolutionary time, see for example, Williams et al. (2017), Dombrowski et al. (2020), Castelle et al. (2021), Martinez-Gutierrez and Aylward (2021), and Aouad et al. (2022) (fig. 1). However, conflicting results regarding the placement of certain putative DPANN clades remain (Feng et al. 2021). Furthermore, it is important to note that the exact placement of the root in the archaeal tree is not yet fully resolved and could be located between two distinct DPANN clades, thus leaving open the possibility that DPANN are paraphyletic (Dombrowski et al. 2020; Aouad et al. 2022). Further analyses, such as the application of gene tree-species tree reconciliations applied to a larger set of representative archaeal genomes will help to test current hypotheses on the early divergence of DPANN. Finally, a reliable interpretation of the early evolution of cellular life, the features of the last universal common ancestor, and the relationship of DPANN and CPR, hinges on the accurate placement of the universal root (Gouy et al. 2015).

## Origin of the Eukaryotic Cell from Prokaryotic Ancestors

The origin of the eukaryotic cell represents one of the most significant and at the same time debated events in life’s evolution. Over the years, a variety of eukaryogenesis models have been put forth, which can be broadly categorized into symbiogenetic and autogenous models, discussed in several comprehensive reviews (Guy et al. 2014; Lopez-Garcia and Moreira 2015; Martin et al. 2015; Koonin 2015a). Although autogenous models assume the vertical evolution of a protoeukaryotic lineage from a root shared with the archaeal and bacterial line of descent, symbiogenetic models suggest that the origin of the eukaryotic cell is a result of a merger of members of at least two distinct microbial lineages belonging to the Archaea and Alphaproteobacteria (Roger et al. 2017; Lopez-Garcia and Moreira 2020) (fig. 1).

Recently, the genomics-based discovery of the Asgard archaea (Spang et al. 2015; Zaremba-Niedzwiedzka et al. 2017) (also referred to as the phylum Asgardarchaeota [Rinke et al. 2021]), has provided important data shedding new light on the origin of the eukaryotic cell. Asgard archaea were originally described to comprise the Loki-, Thor-, Odin, and Heimdallarchaea (Spang et al. 2015; Seitz et al. 2016; Zaremba-Niedzwiedzka et al. 2017), but are now known to include a variety of additional clades (Seitz et al. 2019; Cai et al. 2020; Farag et al. 2021; Liu et al. 2021; Zhang et al. 2021; Wu et al. 2022). Notably, phylogenetic analyses have revealed that the Asgard archaea comprise the closest archaeal sister lineage of eukaryotes (Zaremba-Niedzwiedzka et al. 2017; Liu et al. 2021; Wu et al. 2022) and thereby provided increasing evidence for the evolution of eukaryotes from within the Archaea (Williams et al. 2013, 2020) (fig. 1). But although there is strong support for the monophyly of Asgard archaea and eukaryotes, the exact placement of the eukaryotic branch relative to the various Asgard lineages varies depending on data set composition and evolutionary models used (Zaremba-Niedzwiedzka et al. 2017; Williams et al. 2020; Liu et al. 2021). Expanded sampling of Asgard diversity combined with careful phylogenetic analyses, is likely to provide improved resolution of branching orders and will allow to pinpoint the closest sister-lineage of eukaryotes more precisely.

In agreement with phylogenetic evidence, comparative analyses of the Asgard archaeal genomes have revealed the presence of so-called eukaryotic signature proteins (ESPs) (reviewed in Hartman and Fedorov [2002]; Eme et al. [2017]; Spang et al. [2017]), that is, proteins that were previously thought to be absent from prokaryotic genomes. Notably, these ESPs are homologous to proteins integral to the functioning of complex eukaryotic cells and comprise essential building blocks of the ESCRT (endosomal sorting complex required for transport) system, ubiquitin, trafficking, and informational processing machineries as well as the cytoskeleton (Spang et al. 2015; Zaremba-Niedzwiedzka et al. 2017; Liu et al. 2021). Although the function of these proteins in Asgard archaea remains to be elucidated, the heterologous expression and structural analyses of some of these proteins such as profilins and gelsolins have revealed that they are functionally equivalent to their eukaryotic homologs and suggests that a regulated actin cytoskeleton precedes eukaryogenesis (Akil and Robinson 2018; Akil et al. 2020; Survery et al. 2021).

Because even high quality metagenome assembled genomes (MAGs) (i.e., completeness >90% and contamination <5%, according to Bowers et al. [2017]) usually do not assemble into complete genomes and may contain a low amount of contamination from genomes of other community members or closely related strains, some studies have questioned the reliability of the Asgard archaeal MAGs and in particular raised concerns as to whether ESPs may represent contamination rather than being genuine genomic signatures (Da Cunha et al. 2017, 2018; Garg et al. 2021). However, various lines of evidence during the past years have supported the existence of Asgard archaea, the emergence of the archaeal ancestor of eukaryotes from within this group as well as the presence of ESPs as part of their coding potential: among others, ESPs are encoded within a prokaryotic genomic context, lack introns characteristic of many eukaryotic genes, and are significantly divergent from eukaryotic homologs to exclude contamination (Spang et al. 2015; Zaremba-Niedzwiedzka et al. 2017; Spang et al. 2018). Furthermore, Asgard MAGs have now been reconstructed from a large variety of metagenomes from different environmental samples all over the world and by many different research groups, yet show consistent genomic signatures across the various member clades (Manoharan et al. 2019; Cai et al. 2020; Chen, Wong, et al. 2020; Farag et al. 2020, 2021; Liu et al. 2021; Zhang et al. 2021). Even though the presence/absence pattern of ESPs across Asgard archaea is variable and indicates a complex history of ESP evolution involving duplications, differential loss, and transfers, the shared set of ESPs within specific taxon-level (e.g., class-level) lineages is very consistent and provides strong evidence for ESPs representing genuine signatures of Asgard proteomes (Liu et al. 2021). In line with this, the successful enrichment of the first representative of the Asgard archaea, *Candidatus* Prometheoarchaeum syntrophicum has not only proven the viability of members of this group but also allowed the reconstruction of the first complete genome of a Lokiarchaeote with a characteristic and consistent set of ESPs (Imachi et al. 2020). Finally, initial microscopy analyses have provided insights into the cellular features of extant members of the Asgard archaea including cellular protrusions (Imachi et al. 2020; Avci et al. 2022) and revealed the spatial separation of genomic DNA and ribosomes in certain representatives (Avci et al. 2022).

The analysis of the genomic repertoire of the Asgard archaea has not only enabled predictions of their extant metabolic characteristics but also provided a first baseline to refine symbiogenetic eukaryogenesis models, which predict a syntrophic interaction as an important initial driver for cell–cell interactions (Spang et al. 2019; Imachi et al. 2020; Lopez-Garcia and Moreira 2020; Liu et al. 2021), and represent an extension of the Hydrogen (Martin and Muller 1998) and Syntrophy (Moreira and Lopez-Garcia 1998) Hypotheses. However, more detailed models hinge on resolving the exact placement of the eukaryotic and mitochondrial branches relative to the Asgard archaea (Zaremba-Niedzwiedzka et al. 2017; Liu et al. 2021) and Alphaproteobacteria (Roger et al. 2017; Martijn et al. 2018; Fan et al. 2020; Munoz-Gomez et al. 2022), respectively, as well as the cellular and metabolic features of these ancestors. Additionally, controversies remain with regard to the timing of the events during eukaryogenesis, that is, the timing of the mitochondrial acquisition, the evolution of an endomembrane system as well as the establishment of a nucleus, for example, Baum and Baum (2014), Poole and Gribaldo (2014), Gould et al. (2016), Pittis and Gabaldon (2016), Eme et al. (2017), Tria et al. (2021), Vosseberg et al. (2021) (fig. 1). Finally, the extent to which additional microbial lineages and/or viruses (see below) have contributed to the eukaryotic proteome are still to be determined. Phylogenomics analyses have for example provided support for the hypothesis that the genomic repertoire of eukaryotes was shaped through genetic input from Bacteria other than Alphproteobacteria (Koonin 2010; Rochette et al. 2014; Santana-Molina et al. 2020; Stairs et al. 2020; Hoshino and Gaucher 2021) as well as by viruses, for example, Cermakian et al. (1997), Filée and Forterre (2005), Shutt and Gray (2006), and Harada and Inagaki (2021). Furthermore, a recently proposed updated symbiogenetic model on the origin of the eukaryotic cell has implicated the potential involvement of an additional bacterial lineage (i.e., a Deltaproteobacterium) during eukaryogenesis (Lopez-Garcia and Moreira 2020).

The combination of novel techniques in phylogenetics with cell biological and cultivation approaches (see below) will help to address those conflicting hypotheses of the origin of the complex eukaryotic cell from its prokaryotic ancestors and continue to illuminate the timing of the events during eukaryogenesis (Eme et al. 2017; Roger et al. 2021).

## Eukaryotic Diversity and the Last Eukaryotic Common Ancestor

**Fig. 2.**
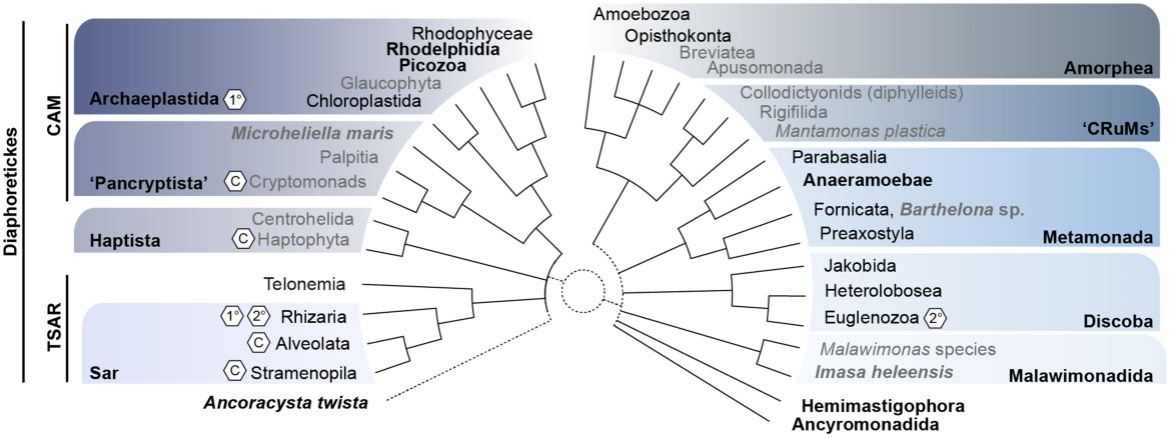
Schematic representation of the phylogenetic diversity of eukaryotes. Groups with taxonomic rankings of phylum level or higher are shown in black (according to Adl et al. 2019 and references in text). Select lineages or organisms that have been recently discovered and placed in the eukaryotic TOL are shown in bold. Eukaryotic supergroups are colored for clarity. Lineages with one or more representative with a primary (1°) secondary (2°) or complex red (C) plastids are indicated with hexagons based on Sibbald and Archibald (2020). Sar, Stramenopila-Alveolata-Rhizaria; TSAR, Telonemia+SAR (Strassert et al. 2019), CAM (Yazaki et al. 2021), Cryptista-Archaeplastida-*Microheiliella maris*; “CRuMs,” Collodictyonids, Rigifilida, *Mantamonas plastica*.

Even though various aspects of eukaryogenesis remain enigmatic, our knowledge of the last eukaryotic common ancestor (LECA) (reviewed in Eme et al. [2017]) and its subsequent diversification has grown substantially in recent years, enabled by a tremendous increase in our sampling of extant eukaryotic diversity. Indeed, although the majority of formally described eukaryotes are multicellular and fall into two phylogenetic groups: Archaeplastida (plants and algae) and Opisthokonta (animals and fungi), it is now clear that the bulk of phylogenetic diversity of eukaryotes is composed of unicellular representatives including “protists” and algae (fig. 2). Major advances in cultivation-dependent (Burki et al. 2020) and cultivation-independent (Burki et al. 2021) methods including symbiosis-aware strategies (Alacid and Richards 2021) for generating sequence data combined with sophisticated bioinformatic tools for genome assembly, gene annotation, and phylogenomic inference have been critical for the genomics-driven exploration of eukaryotic biodiversity. In particular, the last decade has witnessed the discovery of numerous kingdom- and phylum-level lineages and confidently placed those in the eukaryotic TOL (fig. 2), for example, Rhodelphia (Gawryluk et al. 2019), Picozoa (Schön et al. 2021), Anaeramoebae (Stairs et al. 2021), and “CruMs” (Brown et al. 2018) (Collodictyonids, Rigifilids, Mantamonads). Sequence data has also been collected from lineages that have no clear phylogenetic position including *Ancoracysta twista* (Janouskovec et al. 2017), Hemimastigophora (Lax et al. 2018), Ancyromonadida (Torruella et al. 2015), and Malawimonadida (Heiss et al. 2018) that might each represent phylum- (or higher-) level taxonomic ranks.

Supported by these new data, numerous lines of evidence suggest that LECA dated to the Proterozoic (ca. 1.9–1.6 billion years ago) (Parfrey et al. 2011; Eme et al. 2014; Betts et al. 2018) and was characterized by a nucleus and nuclear pores, linear chromosomes with telomeres, genes with spliceosomal introns, complex RNA processing, and regulatory mechanisms, an elaborate endomembrane system (including a Golgi apparatus, endosomes, lysosomes, and peroxisomes), mitochondria, bacterial-type lipids as well as a complex cell cycle (extensively reviewed in Koumandou et al. [2013] and Eme et al. [2017]). Some analyses predict that the LECA proteome was already quite complex with many orthologs (∼10,000) tracing their origin to LECA (Deutekom et al. 2021), though many details regarding components of the various cellular and molecular machineries remain to be further illuminated. One current limitation lies in the unresolved placement of the root in the eukaryotic tree. Depending on gene set and methodology used, the root of the eukaryotic tree has been inferred between Discoba and other eukaryotes (He et al. 2014), between Diaphoretickes + Discoba and Amorphea + CruMs + Malawimonads (Derelle et al. 2015) or between Opisthokonta and all other eukaryotes (Katz et al. 2012; Cerón-Romero et al. 2021). Therefore, the best-studied eukaryotes on which various previous LECA inferences are based, represent derived clades on either side of the putative root: the Archaeplastida within Diaphoretikes and Opisthokonta within Amorphea. It is conceivable that genes conserved in either of these lineages may not necessarily trace their origins back to LECA. For example, a recent review by More et al. (2020) put forth a new term defining hidden ancient homologs as “jotnarlogs” that are shared across eukaryotic biodiversity exclusive of the “model system” lineages. They show that these jotnarlogs are highly relevant for our understanding of the earliest steps in eukaryotic evolution and, among others, comprise proteins mediating fundamentally eukaryotic processes including mitochondrial division (Leger et al. 2015) and membrane trafficking (More et al. 2020). In turn, prospective analyses that make use of the increased sampling of eukaryotic genomic diversity will be crucial to further improve our knowledge on the nature of LECA as well as the root placement in the eukaryotic TOL.

Although most modern eukaryotes share key cellular features, the recent discovery of novel eukaryotic representatives forming distinct branches in the eukaryotic tree have revealed interesting insights into eukaryotic metabolic and cellular diversity. For example, although the alphaproteobacteria-derived mitochondria in extant aerobic eukaryotes house the respiratory chain that couples ATP biosynthesis to the reduction of oxygen, in some anaerobic animals and fungi, the respiratory chain uses alternative electron acceptors to oxygen in order to synthesize ATP, often by “tinkering” with existing cellular systems to synthesize anaerobiosis-specific cofactors or by encoding anaerobiosis-specific proteins (Müller et al. 2012; Gawryluk and Stairs 2021). Further, many anaerobic protists have lost most, if not all, respiratory capabilities and instead couple ATP biosynthesis to fermentative H_2_ production within so-called mitochondria-related organelles (MROs) (Müller et al. 2012; Stairs et al. 2015; Gawryluk and Stairs 2021). Some representatives, such as *Monocercomonoides,* have lost their MROs (Karnkowska et al. 2016), and/or mitochondrial genomes (Stairs et al. 2015) entirely. The genetic origins of the anaerobic metabolism of MROs remains a widely debated topic (see, e.g., Katz 2015; Martin 2017; Leger et al. 2018; Sibbald et al. 2020; Stairs et al. 2020; Tria et al. 2021).

Photosynthesis is a widespread trait across the tree of eukaryotes with representatives in Stramenopila, Alveolata, Rhizaria, Haptista, Pancryptista, Archaeplastida, and Discoba. Primary plastids, derived from the engulfment of an ancestral photosynthetic cyanobacterium with the closest present day relative likely being *Gloeomargarita lithophora* (Ponce-Toledo et al. 2017; Moore et al. 2019), have evolved at least once on the tree of eukaryotes in the Archaeplastida (Sibbald and Archibald 2020) between 2.1 and 1.6 (Sanchez-Baracaldo et al. 2017; Strassert et al. 2021) or 1.8 and 1.1 billion years ago (Betts et al. 2018). There is at least one additional candidate of a primary photosynthetic organelle in eukaryotes in the Rhizarian *Paulinella chromatophora* (Nowack et al. 2008; Nakayama and Ishida 2009). This amoeba houses a specialized organelle called the chromatophore that has its own genome and is thought to have evolved from an ancestral endosymbiont of the *Synechococcus*/*Prochlorococcus* clade (Marin et al. 2005) roughly 90–140 Ma (Delaye et al. 2016). The chromatophore provides a rare opportunity to study the early stages of endosymbiosis having occurring nearly 1 billion years more recently than the primary plastids of Archaeplastida. Other eukaryotes, that is, heterotrophic protists, have acquired secondary or higher order plastids through serial endosymbiosis events, reviewed in Sibbald and Archibald (2020). These higher-order plastids are often surrounded by three or four membranes and, in at least three separate lineages, retain the nuclei (dubbed the nucleomorph) from the engulfed endosymbiotic algae (Sibbald and Archibald 2020). In these cells, there can be as many as four distinct genomes derived from the host nucleus, host mitochondrion, plastid, and nucleomorph. Continued investigations comparing the origin of the gene content and cell biology of these diverse and complex algal lineages as well as phylogenetic and molecular dating approaches will help in identifying the mechanisms necessary for enabling endosymbiosis events and help to further improve our understanding of their timing throughout eukaryotic diversification (Strassert et al. 2021).

## Viruses and the Tree of Life

MGEs are semiautonomous replicative genomic entities that are ubiquitous in the natural environment and believed to be an intrinsic part of cellular evolution (Koonin et al. 2021). They include viruses which may encode one or more proteins comprising the viral particle (virion) encasing the genome of the respective MGE (Koonin et al. 2021). Categorically, viruses are believed to be the most abundant biological entities on the planet, shaping ecological and evolutionary components of the biosphere (Krupovic et al. 2019). The diverse characteristics of MGEs stratify the semiautonomous replicative genomic entities or replicator groups, blurring the boundaries between the major categories within the replicator space, with the Virosphere defined at its core by the *Orthovirosphere*, followed by the *Perivirosphere*, and the remaining replicators falling within the periphery (Koonin et al. 2021).

**Fig. 3.**
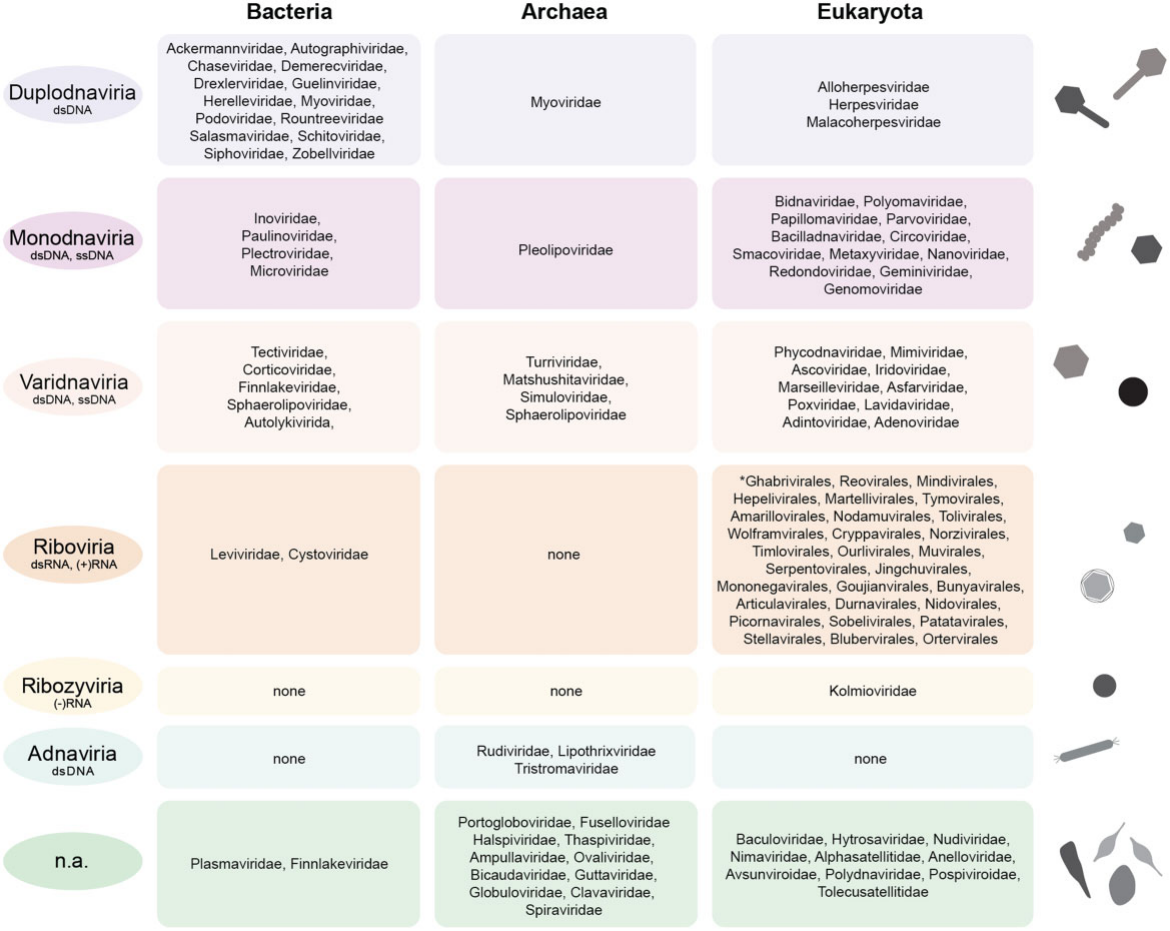
The diversity of the core virosphere and its links to bacterial, archaeal, and eukaryotic hosts. For each viral realm, we depict the diversity of viral families that have representatives infecting members either the Bacteria, Archaea, or Eukaryota, respectively. Asterisk: for eukaryotic viruses assigned to the Riboviria, we report orders instead of families. The shapes represent a small selection of characteristic morphologies seen within certain viral realms. The information on viral families comprising the various realms is derived from the ICTV database (https://talk.ictvonline.org/files/master-species-lists/), that is, ICTV Master Species List 2020.v1.xlsx. (Krupovic et al. 2020; Koonin et al. 2021).

Recent evolutionary insight has classified the core of the virosphere, that is, the *Orthovirosphere*, into six major realms, the *Riboviria*, *Varidnaviria*, *Duplodnaviria*, *Monodnaviria*, *Adnaviria*, and *Ribozyviria* (Koonin et al. 2021), comprising many but not all viral families (figs. 1 and 3). Apart from the *Ribozyviria*, which has been identified in specific vertebrates, all realms are believed to have emerged before or near the origination of the last universal cellular ancestor (LUCA) (Krupovic et al. 2020; Koonin et al. 2021). To fully understand the roles viruses played during the earliest stages of the evolution of cellular life, studies have sought to understand the origins of key viral components. Generally, viral genomes are unified by two core modules: a module that encodes the proteins responsible for genome replication (the replication module) and a module that encodes the proteins that form the virion particle that encapsulates the genome (the morphogenetic module) (Krupovic et al. 2019). Despite great viral diversity, most replication modules can be captured by four hallmark replication protein families: the RNA-dependent RNA polymerase, the reverse transcriptase, the protein-primed family B DNA polymerase, and the rolling-circle endonuclease (Krupovic et al. 2019). All of these share the common ancient RNA-recognition fold and importantly, have minimal to no close sequence identity with replication proteins from cellular organisms. Conversely, investigation into the origins of the capsid proteins that comprise the virion suggests descent from protein families from cellular ancestors, specifically those involved in carbohydrate- or nucleic acid binding (Krupovic et al. 2019). These findings are the foundation of the proposed chimeric model of viral evolution which describes the emergence of the replication module from the primordial replicon pool, with the morphogenetic module evolving on several different occasions through life’s history by acquisitions of structural proteins from hosts (Krupovic et al. 2019). Notably, recent structural and genomics studies into the diversity of archaeal viruses have revealed an abundance of archaea-specific viruses that share no genetic or structural similarity to bacterial and eukaryotic counterparts (Prangishvili et al. 2017; Krupovic et al. 2018) and cannot currently be assigned to any of the viral realms (fig. 3). Beyond unique morphologies across the archaeal viruses, the archaea-specific *Adnaviria* possess a morphogenetic module composed of a capsid protein with a distinct fold not captured by viruses in the other two domains (Koonin et al. 2021). These findings underscore the need for further exploration into the diversity, structure, and function of archaeal viruses.

Viruses and other MGEs are generally not considered part of the TOL (Lopez-Garcia 2012), however the nature of their replication and propagation mechanisms have linked them to critical components of cellular genome dynamics and evolution. Recent efforts have tried to connect the deep origins and diversification of viruses to the earliest transitions in the TOL and diversification of cellular life (Koonin et al. 2020; Weinheimer and Aylward 2020; Irwin et al. 2022). Parasitic replicators play important roles in host-parasite coevolutionary dynamics and the evolution of host genomes (Koonin and Krupovic 2018) and have been placed at the centre of debates regarding eukaryotic evolution and diversification (Koonin et al. 2015; Forterre 2016; Guglielmini et al. 2019; Moniruzzaman, Weinheimer et al. 2020; Collens and Katz 2021; Irwin et al. 2022). Particularly the discovery of eukaryotic NucleoCytoplasmic Large DNA viruses (NCLDVs), also referred to as giant viruses (Raoult et al. 2004), has sparked debates on the boundaries between viruses and cellular organisms as well as raised questions regarding their origins, relationship to cellular life and role in the origin of the eukaryotic cell. NCLDVs comprise members with unique features among viruses including genome sizes that resemble those of some free-living microorganisms, the presence of genes for DNA maintenance including repair, replication, transcription, and translation, complex metabolic capabilities, cytoskeleton components, as well as other signature proteins of complex eukaryotic cells, all of which were originally thought to be confined to cellular life (Schulz et al. 2017; Abrahao et al. 2018; Schvarcz and Steward 2018; Koonin and Yutin 2019; Yoshikawa et al. 2019; Da Cunha et al. 2022; Moniruzzaman, Martinez-Gutierrez et al. 2020; Kijima et al. 2021). Some representatives replicate within viral factories, that is, intracellular compartments in which viral components are localized and that may be enclosed by membranes (Novoa et al. 2005; Suzan-Monti et al. 2007), and can be parasitized by their own virophages (Krupovic et al. 2016). But although those characteristics have originally been suggested to indicate that NCLDVs may form a separate branch within the TOL (Raoult et al. 2004), careful phylogenetic analyses have subsequently shown that NCLDVs have acquired hallmark cellular genes through HGT by their hosts and evolved gigantism multiple times (Williams et al. 2011; Moreira and Lopez-Garcia 2015; Koonin and Yutin 2018; Backstrom et al. 2019), validating the distinction of viruses and cellular life (Moreira and Lopez-Garcia 2009; Lopez-Garcia 2012; Forterre et al. 2014; Koonin and Starokadomskyy 2016). Viruses and in particular NCLDVs have also been hypothesized to have played a role in the origin of the nucleus due to the ability of some representatives to assemble viral factories reminiscent of eukaryotic nuclei (Takemura 2020). However, the direct involvement of a virus in the origin of eukaryotic organellar complexity remains debated (Lopez-Garcia et al. 2017) and viral factories, including those established by certain *Pseudomonas* phages enclosed by a proteinaceous shell (Chaikeeratisak et al. 2017), likely represent analogous structures to eukaryotic nuclei. Nevertheless, viruses and/or MGEs have been found to have shaped the eukaryotic proteome early on including through virus-to-host HGT (Guglielmini et al. 2019; Irwin et al. 2022). For example, the mitochondrial single-subunit RNA polymerase (ssRNAP) has been suggested to be derived from T-odd phages (Cermakian et al. 1997; Filée and Forterre 2005; Shutt and Gray 2006) and eukaryotic telomerases, that ensure the replication of linear chromosomes, are likely derived from a Penelope-like retroelement reverse transcriptase (Koonin et al. 2015). The finding of widespread endogenization of viral genomes, including those of NCLDVs, into eukaryotic host genomes highlights a potentially important strategy underlying virus-to-host HGTs (Feschotte and Gilbert 2012; Moniruzzaman, Weinheimer et al. 2020). Thus, to further disentangle the sources of the eukaryotic proteome and cellular features, prospective phylogenetic analyses benefit from taking into account the wide diversity of viral in addition to prokaryotic genome data (Irwin et al. 2022). In this regard, it is particularly noteworthy that recent metagenomics approaches (some only available as preprints so far) have identified a suite of viruses likely infecting Asgard archaea and belonging to different viral realms (Medvedeva et al. 2021; Rambo et al. 2021; Tamarit et al. 2021; Wu et al. 2022). The genomic and experimental analysis of these and other novel viruses may help to test hypotheses on the features and impact of MGEs in the earliest transitions and diversification of eukaryotic cells.

Taken together, a better understanding of the TOL and major evolutionary transitions hinges on the continued exploration of the virosphere combined with improved phylogenomics and network analyses that allow illuminating the impact of viruses and other MGEs on cellular evolution.

## How to Make Further Progress

Making further progress in our understanding of the TOL and resolving the phylogenetic placement of taxa near key evolutionary branching points requires advances within a wide range of research topics, which we summarize below (Liberles et al. 2020, fig. 4).

**Fig. 4.**
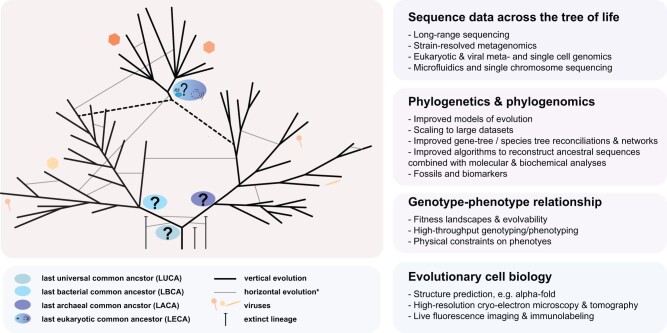
Schematic representation of TOL highlighting key questions and approaches to further illuminate cellular evolution and its connection to viral evolution. See text for more details. Asterisks: please note that horizontal evolution has been estimated to be much more prevalent than indicated in the schematic tree.

### Sequence Data across the TOL

The availability of molecular sequence data for appropriate and extensive taxa sets is a key factor for the reconstruction of congruent phylogenies and understanding life’s evolutionary history in general (Som 2015). Advances in sequencing and data processing techniques have considerably expanded the set of genomes from uncultivated organisms across the TOL and led to a large set of single-cell and metagenome-assembled genomes (SAGs, MAGs) (Eloe-Fadrosh, Ivanova, et al. 2016; Eloe-Fadrosh, Paez-Espino, et al. 2016; Kyrpides et al. 2016; Parks et al. 2017; Gregory et al. 2019). However, the quality of these SAGs and MAGs differs widely (Bowers et al. 2017) and, thus far, rarely provide resolution on single strain level. Current developments of hybrid metagenome assembly methodologies combining both short and long DNA sequence reads (Liao et al. 2019; Wang et al. 2021), innovative genome scaffolding approaches using chromosome conformation capture techniques (Yildirir et al. 2022), and sophisticated (meta)genome assembly computer software (e.g., Bertrand et al. 2019; Kolmogorov et al. 2020; Wang et al. 2021 for review) are promising avenues to obtain high quality strain-resolved MAGs (Chen, Anantharaman, et al. 2020; Olm et al. 2021; Quince et al. 2021) including their CRISPR loci as well as ribosomal RNA operon(s). Such improved metagenomics-driven analyses are also valuable not only for expanding the known diversity of DNA viruses (Paez-Espino et al. 2016; Martinez-Hernandez et al. 2017; Gregory et al. 2019; Moniruzzaman, Martinez-Gutierrez et al. 2020; Bellas and Sommaruga 2021; Edgar et al. 2022), but also to link putative viral genomes to their potential hosts through matching CRISPR spacers (Al-Shayeb et al. 2020); an approach recently used for the identification of viruses infecting Asgard archaea (Medvedeva et al. 2021; Rambo et al. 2021; Tamarit et al. 2021). Considering the complexity of viral populations, a perhaps even more promising approach relies on improved long-range sequencing technologies and was recently used to obtain complete viral genomes without the need for assembly and binning (Beaulaurier, 2020).

In contrast to prokaryotes and viruses, many lineages of eukaryotes, and especially microbial representatives, remain only sparsely sampled, which considerably limits our understanding of the early evolution and diversification of these organisms (Sibbald and Archibald 2017). Only a small number of protists have been enriched in culture and metagenomic approaches targeting uncultivated protists directly are difficult to implement due to the unique and complex genomic features of many representatives (McGrath and Katz 2004), which poses challenges for genome assembly and metagenomic procedures. Further, it should be emphasized that establishing methods for cultivation (or single-cell isolation), nucleic acid isolation, and sequencing from understudied eukaryotes in and of itself is not trivial and requires years of optimization before data analysis can begin (Burki et al. 2020). Many protists harbor symbionts and/or can only be cultivated with other microbes thereby making most protist sequencing projects mini-metagenomics initiatives. Assuming high-quality genomic or transcriptomic data sets can be obtained, the next major obstacle is gene prediction. For genome projects, the nonuniform sequence composition across the genome and the complex architecture of eukaryotic genomes (i.e., large intergenic regions, introns) is a challenge for metagenomic “binning” and gene prediction tools, respectively. Although recent advances in assembling eukaryotic genomes and predicting gene content from complex samples (e.g., nonaxenic cultures or environmental samples) will help in overcoming these obstacles, e.g., West et al. (2018) and Yildirir et al. (2022). Finally, the lack of high-quality reference annotations from diverse eukaryotic representatives, large number of paralogues, and high proportions of lineage or organism-specific putative protein-coding genes in eukaryotic genomes (up to 60% [Karnkowska et al. 2019]) can impede clustering of orthologous groups and poses challenges for the accurate inference of gene history evolution.

### Phylogenetics and Phylogenomics

Ways to resolve incongruences and uncertainties in phylogenies inferred with state-of-the-art phylogenetic and phylogenomic approaches have been reviewed recently (Som 2015; Williams et al. 2021) and will not be extensively discussed. These strategies include, among various others, the development of models of DNA and protein sequence evolution that better capture the processes by which molecular sequences evolve and adequately deal with sources of systematic error (i.e., nonphylogenetic signal) in sequence data: for example, see the recent development of heterotachy mixture models (Crotty et al. 2020). Much of our understanding of the evolutionary history of life mainly derives from analyses of multigene concatenations based on a limited set of universally conserved single-copy marker genes (see, e.g., Martinez-Gutierrez and Aylward 2021; Moody et al. 2022). Elucidating ancient divergences is challenging and requires the use of metrices to assess confidence in tree topologies and bipartitions. However, classical metrices such as the bootstrap, originally designed for single gene trees, have the tendency to overestimate confidence in bipartitions when the analyses are based on long alignments from multigene concatenations (Salichos and Rokas 2013). In turn, it is valuable to explore improved measures to assess confidence in tree and branching patterns (Thomson and Brown 2022), such as, for example, the recently developed internode and tree certainty metrices (Kobert et al. 2016; Martinez-Gutierrez and Aylward 2021). Furthermore, although key to inferring phylogenetic relationships of taxa, multigene concatenations are insufficient to reconstruct the evolution of genomes, which not only results from substitutions but also from gene and genome rearrangements, duplications and the loss and gain of new genes (Long et al. 2013; Andersson et al. 2015). Novel methodologies, capable of capturing simultaneously the vertical and horizontal components of genome evolution such as phylogenetic networks (Dagan 2011), topological data analyses (Chan et al. 2013; Cámara 2017), as well as gene tree-species tree reconciliation methods (Szöllõsi et al. 2012; David and Alm 2011; Szöllosi et al. 2013; Morel et al. 2022), open up new perspectives toward integrating data from viruses, and other genetic elements as well as providing a deeper understanding of gene family evolution including both vertical and horizontal components, across the TOL. For instance, reconciliation methods rely on a model to describe gene tree evolution involving originations, duplications, transfers, and losses under a given species tree and allow to determine the probability of any protein family at any given node in a tree (Williams et al. 2017; Coleman et al. 2021). Furthermore, such approaches can be used to determine the likelihood of certain root positions in the absence of a remote outgroup (Williams et al. 2017; Coleman et al. 2021), which, if available, can cause phylogenetic artifacts such as long branch attraction (Bergsten 2005; Philippe et al. 2005). The modeling of reticulate evolution has recently also been shown to allow dating the TOL (Davin et al. 2018; Wolfe and Fournier 2018), which previously solely relied on the scarce fossil and biomarker record available for the early steps of microbial evolution. Together, this can greatly enhance the understanding and timing of the evolutionary trajectories of life.

### Reconstruction of Ancestral Sequences and Genomes

Progress in the sequencing and assembly of ancient DNA has been successfully applied to reconstruct the genome sequence of organisms (Orlando et al. 2015; Leonardi et al. 2017; Cappellini et al. 2018; Pont et al. 2019) including microorganisms (Arriola et al. 2020; Lammers et al. 2021; Liang et al. 2021) that existed up to hundreds of thousands years ago (i.e., allochronic reconstruction). However, such data is scarce; thus genes, proteins, and genomes of ancestral organisms are predominantly inferred from the sequence of extant taxa using so-called ancestral state reconstruction methodologies (i.e., synchronic reconstruction) (Omland 1999). This includes both ancestral (gene) sequence (Joy et al. 2016; Merkl and Sterner 2016; Gumulya and Gillam 2017; Selberg et al. 2021) and genome reconstruction approaches such as gene tree-species tree reconciliations (see above) (Szöllõsi et al. 2012; David and Alm 2011; Szöllosi et al. 2013; Williams et al. 2017; Coleman et al. 2021; Morel et al. 2022). In turn, features of ancestral organisms and the direction of evolutionary change can be investigated simultaneously.

Progressing further in our knowledge of the features of ancestral organisms involves “resurrecting” those life forms or, at least, some of their proteins (Thornton 2004; Hochberg and Thornton 2017; Mascotti 2022) before characterizing them using molecular, biochemical, and biophysical approaches. Although this has been successfully undertaken for several types of proteins and protein complexes (Finnigan et al. 2012; Shih et al. 2016; Siddiq et al. 2017; Pillai et al. 2020), features of ancestral proteins and protein complexes thought to have played roles in major evolutionary transitions remain largely unknown. In contrast, the “de novo synthesis” of minimal, ancestral cells, still poses significant challenges (Schwille et al. 2018).

### Evolutionary Cell Biology

Reconstructing and understanding the evolution of the ultrastructural complexity of cells and their components throughout the TOL and, most notably, during eukaryogenesis, requires linking gene and genome sequences to protein structures and cellular features. Although the intracellular organization of bacterial and archaeal cells has long been thought to be relatively simple, tremendous advances of microscopy techniques and image analyses now allow probing the cells of these organisms with sufficient resolution to reveal their cytological features in unprecedented detail (Surovtsev and Jacobs-Wagner 2018). Cryoelectron microscopy (Milne et al. 2013) and cryoelectron tomography (Beck and Baumeister 2016; Oikonomou and Jensen 2017) have notably revealed that the ultrastructure of bacterial and archaeal cells is far more complex and diverse than assumed previously (Dobro et al. 2017; Surovtsev and Jacobs-Wagner 2018; Greening and Lithgow 2020; Seeger et al. 2021). Microorganisms are now known to have a wide variety of intracellular organelles (Greening and Lithgow 2020), as well as other intracellular compartments of unknown function including nanospheres and both intracellular and periplasmic vesicles (Dobro et al. 2017). Further, bacterial and archaeal cells often include various types of intracellular filaments, bundles, arrays, and tubes in addition to varied cell appendages (Dobro et al. 2017). The extent to which the cytological features of certain bacteria and archaea, such as *Ca.* P. syntrophicum (Imachi et al. 2020), are related to one another and to those of eukaryotes, remains for now largely unknown considering that genes and proteins involved in their formation have not been identified in many cases. Current advances in the computational prediction of the structure of individual proteins (Baek et al. 2021; Jumper et al. 2021) and both the composition and structure of protein complexes (Baek et al. 2021; Humphreys et al. 2021) have the potential to accelerate the identification of genes involved in protein complexes forming cytological features. Indeed, the accuracy of the protein structures predicted by the neural-network models AlphaFold2 (Jumper et al. 2021) and RoseTTA fold (Baek et al. 2021) rivals that of experimentally determined structures (Baek et al. 2021; Kryshtafovych et al. 2021). Predicted protein structures can help interpreting Coulomb potential maps obtained by cryoelectron microscopy and cellular cryoelectron tomography for the experimental determination of protein structures (Gupta et al. 2021). Furthermore, the development of standards to adequately evaluate the fit of computationally predicted protein models to the Coulomb potential maps of protein complexes may allow to refine protein complex structures and identify genes coding for protein complex components (Masrati et al. 2021). We envision that progress in the computational predictions of protein structures may also allow for the identification of proteins, which share similar folds but little to no amino acid sequence similarity to known components of well-characterized cellular features. Once candidate protein components of a cellular feature of interest have been identified by, for instance, immunogold labeling (Mayhew 2011), the localization, dynamics, and function of the proteins, and corresponding cytological features can be investigated using antibodies conjugated with fluorescent labels and superresolution microscopy (Tuson and Biteen 2015; Möckl and Moerner 2020) as performed, for example, for the analysis of the cytokinesis machinery of bacteria (Holden 2018) and archaea (Pende et al. 2021). Altogether, these protein structure-based approaches combined with high-end microscopy now allow us to bridge the gap between bioinformatic analyses and cell biology and to reconstruct major steps in the evolution of cellular complexity.

### Genotype–Phenotype Relationship

Moving from the reconstruction of the evolutionary history of life to understanding the evolutionary trajectories taken by life forms through time requires clarifying their evolvability (Kirschner and Gerhart 1998; Pigliucci 2008; Payne and Wagner 2019). This includes elucidating the physical constraints on the phenotypes that organisms or their cellular components may take (Alexander 1985; Smith et al. 1985; Arnold 1992; Furusawa and Irie 2020) but also identifying features of biological systems opening opportunities for the emergence of phenotypic variation, innovation, and diversification (Sharov 2014). This emphasizes the need to study fundamental attributes of microbial cells including for example, trade-offs (Garland 2014; Acerenza 2016), allometric scaling laws (West et al. 1997, 2002; Giometto et al. 2013) and robustness (de Visser et al. 2003; Kitano 2007; Masel and Trotter 2010) and their respective underlying causes at the molecular level. Progress in this research area will allow for a better understanding of the relation between genotype and phenotype (i.e., genotype-phenotype map [Pigliucci 2010; Wagner and Zhang 2011; Ahnert 2017]) thereby clarifying the landscape of possible genetic changes. Advances in high-throughput phenotyping and genotyping, targeted genome editing, and single cell approaches (Prakadan et al. 2017; Adli 2018; Ohan et al. 2019; Zahir et al. 2019; Acin-Albiac et al. 2020; Kaster and Sobol 2020; McCarty et al. 2020; Arroyo-Olarte et al. 2021; Rubin et al. 2022), evolutionary synthetic biology (Peisajovich 2012; Baier and Schaerli 2021; Ijäs and Koskinen 2021), and experimental evolution (Van den Bergh et al. 2018), are currently driving progress in the exploration of the genotype–phenotype map. Yet, conceptual, and theoretical developments need to follow technological advances to derive the principles determining the evolution of (micro)organisms. Although such studies are typically conducted on model organisms, a focus on microbial groups placed near key evolutionary branching points would be beneficial for understanding major transitions in the early evolution of life on Earth. This emphasizes the need to isolate and develop laboratory cultivation systems to study members of these microbial groups, most of which remain currently uncultivated (Lewis et al. 2021).

## Conclusion

The TOL is a constantly changing and evolving concept in evolutionary biology, which has helped to depict the vast biodiversity on Earth, including both vertical and horizontal relations of organisms as well as connections to MGEs including viruses. Of course, it will always constitute a simplified illustration of the diversification of life on Earth and can only account for the evolutionary path of extant organisms even though extinct organisms may have contributed to the genetic repertoire of extant genomes. For example, all organisms today are derived from LUCA, yet the early diversification of LUCA was likely shaped by gene influx from now extinct organisms living at the time of LUCA.

Nevertheless, the TOL provides a useful concept for describing and classifying the diversity of organismal life on Earth today (Rinke et al. 2021; Parks et al. 2018) and for improving our understanding of events leading to major evolutionary changes that have dramatically impacted our biosphere. The continuous improvement of analytical, experimental and computational approaches to the study of life’s biodiversity and integration of geological records will further improve our insights into the evolutionary past and allow linking diversification to Earth history. Further, this will help to refine our understanding of evolutionary principles underlying biodiversification, which is crucial for predicting evolution and may help efforts to preserve biodiversity in an ever-changing world.
